# Editorial: Natural compounds/products and livestock productivity: enhancing antioxidant levels, gut health, mitigating greenhouse gas emissions, and disease control

**DOI:** 10.3389/fvets.2025.1664661

**Published:** 2025-08-14

**Authors:** Moyosore Joseph Adegbeye, Valiollah Palangi, Sadarman Sadarman, Abdelfattah Z. M. Salem

**Affiliations:** ^1^Research Centre for Animal Husbandry, National Research and Innovation Agency, Cibinong Science Centre, Bogor, Indonesia; ^2^Department of Life Sciences, Western Caspian University, Baku, Azerbaijan; ^3^Department of Animal Science, Universitas Islam Negeri Sultan Syarif Kasim Riau, Pekanbaru, Indonesia; ^4^Dipartimento di Scienze del Suolo, della Pianta e degli Alimenti (Di.S.S.P.A.), Università degli Studi di Bari, Bari, Italy

**Keywords:** antioxidant, gut health, greenhouse gas, livestock, natural compounds, natural

Growing concerns about antimicrobial resistance, drug residues in animal-derived foods, and the environmental consequences of conventional livestock production have prompted a global shift toward natural alternatives that are safe, multifunctional, and sustainable. Among these, plant-derived bio-actives, microbe-based products, and other natural compounds are emerging as valuable tools to enhance animal health, productivity, and resilience to disease. This shift is particularly relevant given the increasing need to phase out antibiotics as growth promoters and address the ecological pressures linked to intensive livestock systems. This Research Topic brings together 36 articles, comprising original articles, review and meta-analysis selected from 49 submissions, representing contributions from China (31 papers) and five other countries, Japan, Ethiopia, the United States, Korea, and Portugal. The papers span a wide range of livestock species, including ruminants (cattle, sheep, goats, yaks), monogastrics (broilers, layers, ducks, pigeons, geese), and pseudo-ruminants (donkeys), reflecting a global effort to explore natural solutions across diverse production systems.

A key strength of this Research Topic is its integrative perspective. Rather than focusing on single physiological outcomes, the studies address the multifunctional roles of natural compounds, from enhancing antioxidant defenses, modulating gut microbiota, and supporting disease resistance, to reducing methane emissions and improving product quality. This unified framework reflects a growing recognition that sustainable livestock production requires holistic interventions that promote animal health, environmental stewardship, and food safety. The studies can be broadly classified into five categories of natural compounds: including phytogenic compounds, including single-plant extracts, multi-herbal formulations, and essential oils (e.g., *Artemisia annua*, theaflavins, *Pueraria* extract, *Broussonetia papyrifera, Ziziphus spina-christi, Gynura procumbens*, and oregano oil); phyto-microbial systems, which combine plant substrates with microbial fermentation to enhance bioactivity and digestibility (e.g., *Phragmites australis* silage improved with *Bacillus subtilis* and LAB); microbial-derived products, including functional bacteria, yeasts, and bacteriocins (e.g., *Heyndrickxia coagulans* SANK70258, *Rhodotorula mucilaginosa* ZTHY2, and Microcin J25); mineral and vitamin-based compounds, such as Fe-Gly, folic acid, taurine, and vitamin C, that improved metabolism, immunity, and tissue integrity; and oligosaccharides and polysaccharides, including chitosan oligosaccharides and *Lagenaria siceraria* polysaccharides, which play key roles in gut health and immune modulation.

The research themes explored in these papers span several functional domains, performance and antioxidant status (18 papers), immune modulation and gut health (16 papers), reproduction performance and embryo development (six papers), methane mitigation and environmental sustainability (three papers), carcass characteristics and animal product quality (10 papers), and feed ingredient innovation and nutritional strategies (11 papers). Together, these studies reported the potential of natural compounds as viable, regionally adaptable alternatives to synthetic drugs and additives. Many of these compounds are more accessible to smallholder farmers, who play a major role in livestock production across developing and transitional nations.

## Key findings

### Natural products or compounds as ingredients

One of the key future directions in animal nutrition is improving the utilization of unconventional feed ingredients, particularly low-cost, high-fiber resources, to reduce reliance on conventional feedstuff and reduce competition between livestock and human food systems. Integrating *Bacillus subtilis* (BNCC109047) with both homofermentative and heterofermentative lactic acid bacteria enhanced the fermentation quality and nutritional value of *Phragmites australis* (reed), a fibrous and affordable biomass (Liu, Zhang, et al.). This microbial synergy offers a promising strategy for improving the silage quality of lignocellulosic forages, especially in tropical regions where such resources are abundant during the dry season. In a complementary study, Zheng et al. showed that incorporating *Broussonetia papyrifera* silage into ruminant diets improved antioxidant capacity, enhanced mucosal immunity by increasing immunoglobulins and interleukins, and reduced the abundance of potentially pathogenic bacteria such as *Turicibacter* and *Romboutsia* in Kazakh sheep. Therefore, naturally fermented feeds may address feed shortages while promoting gut health and reducing antibiotic dependence.

Feed composition, including bioactive compounds can significantly influence milk quality. In a novel study, Zhou, Huang, et al. reported that fermented *Codonopsis pilosula* residue improved milk yield and metabolite profiles in lactating donkeys. The improvement was linked to immune activation, increased antioxidant capacity, and enhanced glucose and lipid metabolism. This suggest that bioactive residues from medicinal plants could offer dual benefits of improving animal performance while minimizing waste. Global demand for soybeans, largely produced in the Americas, has raised environmental concerns due to its intensive production and transport. While soybean remains an excellent protein source, more sustainable, low-cost alternatives are gaining attention. For example, a soybean-free, low-protein diet comprising rice, rice bran, rice co-product (RCC) meal, sweet potatoes, black tea powder, and tangerine peel powder reduced cost per kilogram of weight gain, increased lean meat yield, reduced cooking losses, and improved pork fatty acid profiles (notably increasing C22:6 and n−3 PUFAs) (Fu et al.). Additionally, this diet lowered the *Firmicutes/Bacteroidetes* ratio and increased the abundance of *Lactobacillus* spp in the colon. This illustrate the potential of unconventional feeds to improve meat quality, gut health and improvement in fiber degrading microbes. However, despite the appeal, long-term use of unconventional feed ingredients often raise questions about their safety, consistency, optimal dosage, and effects on microbiota, animal health, and product quality. Jack et al. (3) noted the nutritional benefits of microalgae like *Spirulina* in ruminants. Yet, the physiological differences between monogastric and ruminant require caution. Spínola et al. found that including *Spirulina* at up to 15% of the diet in broilers impaired weight gain, increased gut viscosity, enlarged digestive tract segments, and altered meat quality by reducing the n-6/n-3 ratio and α-tocopherol content. These effects may be due to their limited ability to digest microalgal cell walls, particularly at young age. Thus, while unconventional feed ingredients from fibrous forages and plant residues to microalgae offer great potential to reduce costs and improve animal health, careful consideration of species-specific responses, inclusion levels, and long-term effects remain essential. Continued research in microbial fermentation, bioactive compound profiling, and gut microbiome interactions will be key to unlocking their full value in modern animal production.

### Natural products on stress and animal welfare

Stress is an unavoidable aspect of livestock production, triggered by physical exertion, transportation, extreme temperatures (especially heat), overcrowding, and intensive management. Many of these factors lead to oxidative stress at the cellular level, depleting antioxidant defenses, weakening immune function and increasing free radical production (1). Free radical levels can rise due to aging, infections, injuries, poor diet, or environmental disruptions, further amplifying oxidative stress. Transportation, whether from hatcheries to farms or farms to markets and slaughterhouses is a key contributor to physiological stress in animals. In yaks, for example, transport stress was linked to body weight loss and elevated plasma concentrations of lactate dehydrogenase, creatine kinase, malondialdehyde (MDA), cortisol, and lipopolysaccharides, all of which are biomarkers of oxidative and inflammatory responses. However, rumen-protected glucose (RPG) combined with taurine (TAU) supplementation minimized post-transport weight loss, improved antioxidant capacity, and enhanced immune function, effectively alleviating stress responses in yaks (Wang, Zhao, et al.). In poultry, similar stress mitigation strategies are gaining attention. Qu et al. demonstrated that beyond classical antioxidants like selenium, zinc, and vitamins C and E, a compound herbal extract containing *Astragalus, Epimedium*, and *Fructus Ligustri Lucidi* (AEF) enhanced immune function and antioxidant status in goslings. AEF improved intestinal morphology (as seen in better villus height to crypt depth ratios), reduced the expression of inflammatory markers, and improved meat quality under peroxide-induced oxidative stress. This suggest broader applications of such compounds in other avian monogastric.

Beyond environmental stress, certain physiological stages, such as the perinatal period, place additional demand on high-yielding dairy cows. During this phase, bovine mammary epithelial cells (BMECs) experience accelerated metabolism and generate excess reactive oxygen species (ROS), contributing to oxidative damage and increasing susceptibility to mastitis, a prevalent and economically costly disease affecting milk yield and quality. Baicalin, a flavonoid derived from *Scutellaria baicalensis*, exhibited potent antioxidant, anti-inflammatory, and anti-apoptotic effects. It regulated oxidative stress-induced cell apoptosis in BMEC challenged with hydrogen peroxide (H_2_O_2_), offering a potential natural therapeutic alternative to manage periparturient oxidative stress (Kong et al.). In clinical mastitis, inflammation of the mammary gland is typically due to microbial infection or physical injury. Conventional management often involve minimizing pathogen exposure or injecting antibiotics directly into the udder, which carries the risk of residue accumulation (4). A promising alternative is the use of *Macleaya cordata* extract, which was reported to improve immune indices and reduce somatic cell count (SCC), serum amyloid A (SAA), and endotoxins in cows with mastitis, without negatively impacting milk production (Khattab et al.). Another critical stressor is weaning, particularly in neonatal piglets transitioning from milk to solid feed. This phase is often associated with diarrhea, inflammation, oxidative stress, and growth depression. Common interventions include zinc oxide and antibiotics, which face increasing regulatory restrictions due to environmental and resistance concerns. In this context, taurine, a naturally occurring amino sulfonic acid with antioxidant properties, has emerged as a promising alternative. It reduced diarrhea rates, enhanced intestinal barrier function, and alleviated oxidative and inflammatory damage in weaned piglets via activation of the Nrf2/HO-1 signaling pathway (Zhou, Wu et al.).

### Natural products on Gut health and Immune functions

With the global ban on indiscriminate use of antibiotics as growth promoter in livestock production, producers and researchers are increasingly seeking safe, effective alternatives to maintain animal health and productivity. Gao Y. et al. emphasized that optimizing feed formulations and exploring novel, local, eco-friendly additives like *Atractylodes lancea* have become critical. These natural compounds show promise in improving performance, immunity, reducing heat stress, and supporting gut health. Gut-related issues in livestock are often linked to imbalances in the gut microbiota, weakened mucosal barriers, or inflammation. A key component of gut health is the function of Trefoil Factor Family (TFF) proteins, which help protect the gastrointestinal lining and regulate immune responses and it was recommended that feed additives promoting TFF protein synthesis could serve as natural alternatives to antibiotic growth promoters, helping to preserve gut integrity and resist infections (Fasina et al.). A recent study by Zhang Z. et al. demonstrated that polysaccharides extracted from *Lagenaria siceraria* (Molina) Standl. (LSP) helped regulate gut microbiota composition and increased the production of beneficial short-chain fatty acids. These changes contributed to improved intestinal immunity, better development of immune organs, and enhanced growth performance in broiler chickens. The small intestine, especially the jejunum, is the primary site for nutrient absorption in livestock, due to its high osmotic pressure (2). When its functions are compromised, animal health and growth can be severely affected. To prevent/manage this, Gang et al. found that water extracts of *Artemisia annua* improved immune function and antioxidant capacity in the small intestines of lambs, and also upregulated genes linked to mucosal health. One major challenge of indigenous pigs is their relatively slow growth, which often lead to increased reliance on antibiotics to prevent disease or as growth promoter. However, Wu H. et al. reported that *Pueraria* extracts offered a natural alternative by boosting beneficial microbes like *Bacillus acidi lactici* and *Saccharomycetes*, while reducing harmful *E. coli* and *Salmonella* in the jejunum. These shifts improved growth, feed efficiency, and immune responses, including higher immunoglobulin M and stronger intestinal barrier function.

While antibiotics have traditionally been added to livestock water or feed to support growth and health, increasing restrictions and concerns about resistance have prompted the exploration of natural alternatives. One promising example is fermented Shuanghuanglian, an herbal blend made from Honeysuckle flower, Baical skullcap root, and *Fructus forsythiae*. According to Xu Y. et al., adding 0.5% of this mixture to the drinking water of laying hens significantly improved their production performance, offering a natural way to support commercial poultry farming. Likewise, black tea-derived theaflavins (TF) enhanced laying performance, antioxidant capacity, yolk color, and lipid metabolism in hens (Zhou L. et al.), offering a natural way to improve egg quality. These improvements could reduce reliance on artificial yolk colorants and enhance overall egg quality in commercial operations. In broilers, ginseng stem-leaf extract has also shown promise. As reported by Zhang P. et al., supplementation improved growth performance and meat quality while enhancing antioxidant status, immune function, and cholesterol metabolism to varying degrees. Among ducks, low immunity remains a persistent challenge that contributes to high mortality rates. Addressing this, Ai et al. found that adding *Gynura procumbens* extract to duck feed significantly increased serum total antioxidant capacity, improved immune responses, and enhanced meat quality, offering hope for healthier, more productive duck farming.

Essential oils (EOs) are another natural tool with antimicrobial and performance-boosting benefits. Li et al. in their meta-analysis showed that EOs in calf diets improved milk production and beta-hydroxybutyric acid levels without disrupting rumen function. Oregano essential oil (OEO), in particular, reduced excessive immune responses and intestinal damage in Holstein bulls (Xu M. et al.), suggesting a role in supporting gut health and immunity. For newborn calves with underdeveloped digestion, Zhang M. et al. found that a compound herbal extract (CHE) made from Honeysuckle, Astragalus, Magnolia bark, and Tangerine peel improved digestion, immunity, microbiota balance, and growth, without toxic effects.

### Microbial

*Heyndrickxia coagulans* SANK70258, a Gram-positive bacterium, improved the growth performance of pigs, particularly during the lower-risk growing phase (Aida et al.). Microcin J25 (MccJ25), an antimicrobial peptide and a stable bacteriocin produced by *Escherichia coli* improved systemic metabolism, enhanced antioxidant defenses, strengthened intestinal barrier integrity and ultimately promotes pigeon health and survival (Cao et al.). This study support the application of MccJ25 as a functional feed additive in poultry production. Similarly, *Rhodotorula mucilaginosa* ZTHY2, a marine yeast strain, also improved the growth performance of ducks by enhancing immune function and antioxidant capacity. It upregulated serum components such as C3, C4, IgG, and cytokines (IFN-γ, IL-2, IL-4, IL-6, TNF-α), and modulated their gene expression in key immune organs (Wu J. et al.).

## Natural products/compounds on sustainability

### Methane reduction

Accurate methane emission data from ruminant production systems in Africa remain limited, primarily due to the high cost and limited availability of advanced measurement equipment such as Laser methane detector, GreenFeed, SF6, and respiratory chamber. Nevertheless, a consistent pattern emerging from reports is that ruminants in Africa tend to have high methane emission intensity, often attributed to the fibrous nature of native forages, widespread reliance on crop residues and agricultural by-products, and generally low product output (weight gain and milk yield). To address this challenge, Bature et al. reported on the potential of some locally available phytogenic feed additives, including trees, herbs, shrubs, and tannin-rich forages, as sustainable tools for reducing methane emissions in ruminants. In Ethiopia, Bekele et al. demonstrated that Menz sheep fed test diets containing *Acacia nilotica* and *Ziziphus spina-christi* leaves, either alone or in combination with brewery spent grains (BSG), exhibited a methane reduction of up to 67%. The methane-reducing effect was largely attributed to the condensed tannins present in these forages, which are known to modulate rumen fermentation and suppress methanogenesis. Similarly, in Korea, Bharanidharan et al. showed that when *Pharbitis nil* seeds were used in ruminant diets, there was up to 17.2% decrease in methane yield (g/kg OM), alongside a 7.6% reduction in urinary nitrogen excretion. In addition, the inclusion of *P. nil* increased metabolizable energy intake by 14.7% (as a percentage of gross energy intake) and reduced rumen protozoa population**s**, particularly *Entodinium caudatum*, by 40%, further supporting its anti-methanogenic and eco-friendly properties. These show the potential of locally available, plant-based feed additives as practical and cost-effective strategies for methane mitigation, particularly in low-input systems where conventional feed technologies may not be feasible. Apart from reduced emissions, this approach also offers benefits for nutrient utilization and productivity, contributing to both environmental sustainability and livestock development goals across diverse agro-ecological regions.

### Nutrient excretion reduction

Excessive use of inorganic trace elements in livestock feed is common to meet nutritional needs, but it often leads to gastrointestinal stress and high levels of excretion, which can harm the environment. For piglets, iron injections are routinely given after birth, yet unused iron in the gut can be taken up by harmful microorganisms. Organic iron sources, such as iron glycinate (Fe-Gly), offer better digestibility and absorption. Supplementing piglet diets with 50 mg Fe-Gly has been shown to significantly improve feed intake, daily weight gain, and reduce diarrhea rates by 40%. It also enhanced iron status by increasing serum total iron-binding capacity, indicating more efficient iron transport. Fe-Gly helped protect against enterotoxigenic *Escherichia coli* (ETEC)-induced intestinal damage by improving jejunal morphology and regulating genes linked to gut health. This suggest that Fe-Gly not only meets the piglets' iron needs but also limit iron availability to pathogens like ETEC, supporting gut health under infectious stress (Gao Q. et al.). In finishing pigs, replacing all inorganic trace elements with up to 70% organic alternatives maintained performance, improved antioxidant capacity, reduced fecal excretion, enhanced gut health and microbiota diversity, and presented a more sustainable approach for modern pig production (Xu et al.).

### Reproductive health

Reproduction remains one of the most sensitive and performance-defining processes in livestock systems. Recent studies have demonstrated that natural compounds and microbial derivatives can profoundly impact reproductive health and efficiency, particularly during critical windows such as late gestation, lactation, and early development. Maternal microbe-derived antioxidants (MA) supplementation increased litter size at weaning, improved milk quality (higher dry matter and fat), and reduced oxidative stress markers (Tang et al.). Furthermore, piglets born to these sows had better post-weaning growth and immunological resilience, emphasizing the maternal diet's programming effect. The antioxidant-rich MA compounds fermented mixtures involving *Rosa roxburghii*, sea buckthorn, and probiotics, enhanced redox balance and shifted gut microbial profiles in both dams and offspring. In a complementary approach, Zhao et al. evaluated a composite plant extract supplement, ALAEm, during late gestation. Derived from nine traditional Chinese medicinal herbs, ALAEm improved live piglet births, placental vascular development, and reduced inflammatory markers, linked to upregulation of nutrient transport genes and antioxidant defenses in the placenta. These outcomes support the role of botanical extracts in reducing gestational stress and improving uteroplacental efficiency, a key bottleneck in sow productivity under antimicrobial-free systems. Similarly, folic acid improved folate status in eggs and offspring and modulated ovarian gene expression linked to reproductive competence in broiler breeders. These molecular effects, especially in Wnt signaling and steroid biosynthesis pathways, indicate folic acid regulatory role beyond basic nutrition. Du et al. explored *in ovo* vitamin C delivery in broilers, showing accelerated hatching, improved yolk absorption, and better hepatic metabolic indicators. Though studied in avian models, these results have relevance for prenatal antioxidant support in mammals under suboptimal conditions. Research evidence show that chitosan oligosaccharide (COS) can mitigate intrauterine growth restriction (IUGR), a persistent challenge in pig production. COS supplementation reduced stillbirth and mummified fetuses, and enhanced placental transporter expression and VEGFA signaling. These improvements translated into better gut function, redox status, and immune parameters in IUGR piglets, reinforcing the placenta-gut-health axis in neonatal survival and performance (Wang, Fang, et al.). The adoption of natural compounds such as MA, folic acid, COS, plant extracts, and vitamins is no longer an experimental novelty; they represent scalable, sustainable tools to enhance reproductive efficiency while reducing reliance on synthetic additives and antibiotics.

### Others

Study by Liu, Liao, et al. on the physicochemical profiles of mixed rumen microbes (MRM), showed how surface tension (ST) and specific surface area (SSA) of rice straw-derived neutral detergent fiber (NDF) influence rumen microbial function and fermentation efficiency. The result showed those physical characteristics of the fermentation environment such as substrate structure and liquid surface tension, play a pivotal role in modulating microbial behavior, fermentation outcomes, and gut homeostasis. The study adds a novel dimension to our understanding of how physical feed properties and medium conditions can be manipulated to optimize rumen function, particularly in fiber-rich diets.

## Conclusions

A promising future direction is the exploration of phyto-microbe synergy, harnessing the combined benefits of plant metabolites and microbial products to improve animal health and productivity while ensuring safety and sustainability. The botanical and microbial biodiversity found across tropical, arid, semi-arid, temperate, and subtropical zones offers an untapped reservoir of bioactives with localized benefits. However, further research is needed to identify active ingredients, determine effective dosages, and evaluate the long-term use of these compounds to ensure consistency and efficacy. This editorial collection marks a step forward in aligning livestock research with One Health and eco-efficient production goals, providing a scientific basis for the responsible use of natural compounds in livestock feeding systems.
